# Synthetic microbial consortium enhances acetoin production and functional quality of citrus vinegar via metabolic and process optimization

**DOI:** 10.3389/fmicb.2025.1664794

**Published:** 2025-08-13

**Authors:** Yerui OuYang, Shaoqing Zou, Panpan Liu, Linfeng Xie, Yiwen Xiao, Ya Wang, Guangjie Wu, Jiantao Liu, Bin Liu, Boliang Gao, Du Zhu

**Affiliations:** ^1^Key Laboratory of Natural Microbial Medicine Research of Jiangxi Province, College of Life Sciences, Jiangxi Science and Technology Normal University, Nanchang, China; ^2^Key Laboratory of Microbial Resources and Metabolism of Nanchang City, College of Life Sciences, Jiangxi Science and Technology Normal University, Nanchang, China; ^3^Microbial Resources Innovation and Application Experimental Center, College of Life Sciences, Jiangxi Science and Technology Normal University, Nanchang, China; ^4^Key Laboratory of Protection and Utilization of Subtropic Plant Resources of Jiangxi Province, Jiangxi Normal University, Nanchang, China; ^5^East China University of Technology, Nanchang, China

**Keywords:** acetoin biosynthesis, microbial combination, *Lactobacillus plantarum*, *Acetobacter pasteurianus*, transcriptome analysis, fruit vinegar, co-culture fermentation, functional metabolites

## Abstract

Acetoin (3-hydroxy-2-butanone) is a key flavor compound that enhances the sensory profile of fruit vinegar. In this study, we developed a high-yield acetoin fermentation process using a synthetic microbial combination composed of *Lactobacillus plantarum* NF2 and *Acetobacter pasteurianus* NF171 for citrus vinegar production. By screening compatible strains and optimizing fermentation parameters, the co-culture system significantly improved acetoin synthesis compared to single-strain fermentations. Transcriptome analysis revealed that the consortium facilitated acetoin production by downregulating NADH metabolic flux and upregulating transcription of the α-acetyllactate synthase gene. Under optimized conditions—including 3% inoculum size, 120 rpm agitation, 33°C temperature, and 20 g/L sugar supplementation—the acetoin concentration reached 4033.72 ± 64.48 mg/L, representing an eight-fold increase over monoculture conditions. In addition to flavor enhancement, the process also enriched the vinegar with functional components, including acetic acid (57.91 ± 0.82 g/L), phenolic acids (such as chlorogenic and ferulic acids), and flavonoids (such as rutinarin and nobiletin). These compounds contribute to both product stability and potential health benefits. This study provides a practical and scalable strategy for enhancing acetoin biosynthesis and improving the quality of functional fruit vinegar through rational design of microbial consortia and process engineering.

## Graphical abstract

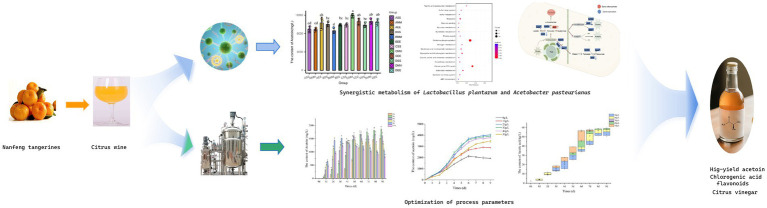

## 1 Introduction

Food fermentation, humanity’s oldest biotechnological practice, involves microbial communities transforming substrates through metabolic network reconstruction (Rau and Zeidan, 2018). Traditional fermentation relies on spontaneous environmental microorganism succession, which, despite creating flavor diversity, faces challenges like inconsistent microbial succession and redundant metabolic pathways. These issues hinder product standardization and functional enhancement, especially in industrial production (Chen et al., 2023). The integration of microbiome and synthetic biology technologies has led to “Microbiome Engineering,” shifting food fermentation from experience-based to rational design. This approach focuses on analyzing metabolic interactions within natural fermentation microbial communities and achieving targeted compound synthesis by regulating carbon and nitrogen fluxes at key nodes (Walsh et al., 2023). Within this technical framework, the construction strategy for synthetic microbial communities draws upon the characteristics inherent in natural fermentation microbial communities. By meticulously controlling the composition and functionality of microorganisms, this approach markedly enhances the quality stability and safety of fermentation products (van Leeuwen et al., 2023). Notable applications include the elimination of harmful by-products, such as ethyl carbamate, through the optimization of metabolic interactions (Pang et al., 2023), and the enhancement of flavor compound synthesis, such as esters, via quorum sensing regulation (Gu et al., 2022). It is noteworthy that the synergistic system of lactic acid bacteria (LAB) and acetic acid bacteria (AAB) has emerged as an exemplary model for investigating the metabolic division of labor in microorganisms, owing to its multifaceted roles in acidification regulation, flavor generation, and pathogen inhibition (Xia et al., 2022). *Acetobacter pasteurianus* NF171 (AAB) makes specific contributions to flavor formation through multiple metabolic pathways, synergizing with *Lactobacillus plantarum* NF2 (LAB) in the citrus vinegar fermentation system of this study: as a major metabolic product, acetic acid generated by *Acetobacter pasteurianus* NF171 (reaching up to 68.13 g/L in the DSS group, Table 1) not only imparts the characteristic sourness to vinegar, forming the flavor base, but also influences the perception of other flavor substances by regulating the acidity of the system; Intermediate products such as acetaldehyde produced through its ethanol oxidation pathway can interact with substances like acetoin generated by LAB (reaching 4033.72 mg/L under optimized conditions) to form volatile aroma components such as esters or aldehydes, enriching characteristic aromas like cream and nuts; meanwhile, *Acetobacter pasteurianus* NF171 regulates pH through metabolic activities, ensuring the stability and solubility of phenolic acids (e.g., chlorogenic acid in the DEE group reaches 14.92 mg/L, Table 1) and flavonoids (e.g., naringin in the BMM group reaches 92.1 mg/L, Table 2), thereby enhancing flavor complexity; In addition, its regulation of the NADH/NAD^+^ redox balance can reduce the over-oxidation of acetoin, maintaining the accumulation of key flavor substances. In summary, AAB not only directly participates in flavor formation through acid production, but also synergistically shapes the unique sensory profile of citrus vinegar by regulating the chemical environment, interacting with LAB metabolites, and stabilizing active components. In the context of multi-strain relay fermentation, the enhancement of citrus fruit vinegar quality necessitates a paradigm shift from the optimization of traditional processes to the exploration of multi-strain metabolic network interactions. Among them, acetoin (3-hydroxy-2-butanone), as a precursor compound of furanone aroma substances, has the aroma characteristics of cream, caramel and nuts, and is a key contributor to the characteristic flavor substances in vinegar (Wätjen et al., 2023).

**Table 1:** Types and contents of total acid.

Group	ASS	AMM	AEE	BSS	BMM	BEE	CSS	CMM	CEE	DSS	DMM	DEE
Oxalic acid	446.96 ± 22.64bc	476.24 ± 17.11b	376.23 ± 11.99def	329.84 ± 20.13 g	442.98 ± 18.37bc	409.97 ± 20.41 cd	468.23 ± 10.82b	391.70 ± 19.50de	527.83 ± 10.09a	222.65 ± 17.21 h	377.02 ± 9.07def	357.36 ± 20.79efg
Malic acid	261.53 ± 10.18 cd	221.53 ± 14.99e	357.96 ± 17.40b	368.43 ± 17.35b	408.69 ± 19.51a	376.70 ± 13.66b	214.16 ± 2.81e	162.80 ± 9.35 g	170.03 ± 12.96 fg	209.00 ± 15.47e	252.47 ± 10.82d	196.38 ± 9.61ef
Vitamin C	135.45 ± 6.60cdef	149.10 ± 7.05bc	145.65 ± 6.71bcd	138.37 ± 14.50cde	132.90 ± 3.61def	124.90 ± 7.01ef	72.50 ± 2.05 g	120.93 ± 3.12f	126.68 ± 1.02ef	79.74 ± 3.55 g	78.51 ± 3.57 g	136.01 ± 8.53cdef
Lactic acid	252.13 ± 22.07fgh	299.21 ± 14.71 fg	212.40 ± 14.88gh	531.78 ± 14.21 cd	431.23 ± 17.14de	334.90 ± 31.47efg	1216.74 ± 82.59a	959.44 ± 172.54b	468.17 ± 83.17d	246.15 ± 10.52fgh	163.69 ± 13.93 h	271.02 ± 16.07fgh
Acetic acid	53007.87 ± 2761.78e	47610.16 ± 678.30f	48211.33 ± 825.86f	53842.21 ± 701.79e	62080.19 ± 1798.11b	57383.17 ± 871.01 cd	55176.61 ± 975.18de	61402.19 ± 849.60b	52670.46 ± 1989.53e	68126.38 ± 1187.38a	65774.76 ± 1155.08a	60285.66 ± 1704.21bc
Maleic acid	2.24 ± 0.13c	1.24 ± 0.03e	0.00 ± 0.00f	0.00 ± 0.00f	0.00 ± 0.00f	0.00 ± 0.00f	0.00 ± 0.00f	1.19 ± 0.10e	0.00 ± 0.00f	2.30 ± 0.15c	1.68 ± 0.49d	1.56 ± 0.24de
Citric acid	3446.10 ± 38.90j	4051.81 ± 11.35ef	3828.02 ± 72.54gh	3476.63 ± 8.49j	3927.95 ± 48.19efgh	3967.53 ± 82.47efg	3862.05 ± 92.02fgh	3606.79 ± 157.56ij	3750.01 ± 41.59hi	4073.04 ± 126.77e	4962.57 ± 92.43c	4975.31 ± 112.80c
Succinic acid	1378.31 ± 39.24de	1553.75 ± 18.11bc	709.50 ± 72.36 fg	1441.95 ± 62.88 cd	1675.18 ± 98.46b	1281.17 ± 63.47e	329.92 ± 18.08ij	451.15 ± 12.60i	601.82 ± 7.14gh	825.50 ± 36.76f	1312.56 ± 42.40e	1277.91 ± 53.88e
Fumaric acid	10.08 ± 0.53ab	11.07 ± 0.78a	7.90 ± 0.18 cd	9.75 ± 0.64b	8.37 ± 1.07c	3.44 ± 0.37 g	5.36 ± 0.52ef	4.60 ± 0.27f	2.90 ± 0.09 g	3.46 ± 0.16 g	4.87 ± 0.56ef	2.36 ± 0.05 g
Protocatechuic acid	3.80 ± 0.52bc	3.88 ± 0.15bc	5.01 ± 0.33a	3.34 ± 0.26 cd	3.77 ± 0.15bc	2.93 ± 0.04d	4.00 ± 0.16b	3.83 ± 0.21bc	2.87 ± 0.09d	4.01 ± 0.24b	4.96 ± 0.65a	3.03 ± 0.14d
4-Hydroxybenzoic acid	5.13 ± 0.64ab	4.80 ± 0.47abcd	4.85 ± 0.44abcd	4.56 ± 0.07bcde	4.00 ± 0.15ef	4.11 ± 0.26def	4.04 ± 0.25ef	4.25 ± 0.29cdef	4.70 ± 0.22abcde	4.93 ± 0.24abc	4.80 ± 0.17abcd	3.67 ± 0.13f
Chlorogenic acid	12.07 ± 0.31 cd	9.82 ± 0.33f	10.26 ± 0.73ef	12.45 ± 1.19 cd	11.48 ± 0.81de	9.14 ± 0.47f	13.41 ± 0.64abc	12.15 ± 0.90 cd	13.49 ± 0.75abc	14.42 ± 0.58ab	9.96 ± 0.21f	14.92 ± 0.40a
Caffeic acid	8.82 ± 0.44f	9.46 ± 0.61ef	10.05 ± 0.90ef	10.36 ± 1.31def	10.06 ± 0.59ef	12.87 ± 0.68a	8.73 ± 0.54f	9.72 ± 0.57ef	11.15 ± 0.39bcde	10.00 ± 0.68ef	9.86 ± 0.34ef	12.68 ± 0.56ab
p-Coumaric acid	7.48 ± 0.48ab	6.77 ± 0.13bcde	6.32 ± 0.57cde	6.83 ± 0.57bcd	5.97 ± 0.33de	6.24 ± 0.31 cde	5.73 ± 0.53e	7.27 ± 0.50abc	6.05 ± 0.59de	7.57 ± 0.19ab	6.87 ± 0.40bcd	6.57 ± 0.36bcde
Ferulic acid	11.60 ± 0.29a	10.84 ± 0.43abc	10.23 ± 0.69c	10.46 ± 0.79bc	10.85 ± 0.41abc	8.93 ± 0.59d	8.61 ± 0.24d	10.68 ± 0.41abc	9.02 ± 0.50d	11.40 ± 0.43ab	10.62 ± 0.37abc	10.50 ± 0.50bc
Sinapic acid	5.70 ± 0.17a	5.11 ± 0.22bcd	5.36 ± 0.15abc	5.36 ± 0.24abc	4.46 ± 0.12e	4.96 ± 0.42cde	3.83 ± 0.25f	5.60 ± 0.35ab	4.80 ± 0.17cde	4.69 ± 0.18de	5.63 ± 0.26ab	4.91 ± 0.10cde

Data shown are the mean ± SD of triplicate, values with different Roman letters in the same row indicating significant differences at *p* < 0.05 (Duncan’s test).

**Table 2:** Types and contents of flavonoids.

Group	Eriocitrin	Rutinarin	Naringin	Neohesperidin	Naringenin	Nobiletin
ASS	5.85 ± 0.31c	69.34 ± 5.20a	66.9 ± 2.98cde	20.55 ± 1.04ab	6.23 ± 0.11bcd	4.36 ± 0.11abc
AMM	7.79 ± 0.07a	61.94 ± 3.29b	90.1 ± 4.69a	20.88 ± 0.76ab	6.52 ± 0.29b	4.22 ± 0.29abcd
AEE	5.20 ± 0.14d	51.83 ± 0.87defg	77.1 ± 4.23b	18.58 ± 0.97bcde	5.99 ± 0.27cde	3.58 ± 0.27f
BSS	3.93 ± 0.13ef	52.63 ± 0.78def	82.2 ± 2.41b	19.33 ± 1.27bcd	6.28 ± 0.19bcd	4.01 ± 0.19bcdef
BMM	4.48 ± 0.35ef	51.42 ± 5.20defg	92.1 ± 0.89a	16.32 ± 0.62efg	6.31 ± 0.11bcd	4.33 ± 0.11abc
BEE	4.21 ± 0.12ef	44.72 ± 5.48 g	64.4 ± 0.46cde	17.79 ± 0.86cdef	5.36 ± 0.17f	4.55 ± 0.17a
CSS	5.56 ± 0.18 cd	46.04 ± 3.58efg	53.6 ± 1.07 g	16.22 ± 0.41efg	6.46 ± 0.34bc	4.59 ± 0.34a
CMM	6.92 ± 0.65b	54.44 ± 3.06 cd	62.4 ± 3.12def	14.01 ± 1.19 g	5.90 ± 0.20de	4.53 ± 0.20a
CEE	5.92 ± 0.46c	36.73 ± 2.18 h	70.6 ± 4.54c	17.03 ± 0.59def	5.59 ± 0.35ef	4.44 ± 0.35ab
DSS	7.53 ± 0.25a	60.52 ± 1.26bc	63.0 ± 2.36de	20.70 ± 1.40ab	5.39 ± 0.29f	4.24 ± 0.29abcd
DMM	6.89 ± 0.29b	54.60 ± 1.12 cd	61.4 ± 0.79ef	16.10 ± 1.41efg	6.18 ± 0.16bcd	3.87 ± 0.16cdef
DEE	4.14 ± 0.30ef	45.61 ± 1.65 fg	63.5 ± 2.00de	17.14 ± 0.72def	6.28 ± 0.25bcd	3.77 ± 0.25def

Data shown are the mean ± SD of triplicate, values with different Roman letters in the same row indicating significant differences at *p* < 0.05 (Duncan’s test).

The biosynthesis of acetoin represents an emergent property of microbial carbon metabolism, governed by the multi-dimensional regulation of “growth-differentiation-stress response” (Wang et al., 2025). The core metabolic pathway is initiated by the condensation of pyruvic acid, catalyzed by α-acetyllactate synthase (ALS), leading to the formation of the key intermediate α-acetyllactate (AL) (Zuljan et al., 2014). This intermediate is subsequently converted into acetoin, diacetyl (DA), and 2,3-butanediol (BD) through both enzymatic and non-enzymatic pathways (Guo et al., 2012). This metabolic network is characterized by a highly dynamic equilibrium. The efficiency of the AL decarboxylase (ALDC) enzyme in catalyzing the primary pathway to produce (3R)-acetoin is modulated by pH (optimal range 6.0–6.5) and the level of rotational speed (Zheng et al., 2023). The reversible reaction between acetoin and BD, mediated by BDH, along with the irreversible reduction of DA, forms a competing branch within the pathway (Zheng et al., 2023). Microorganisms achieve directional distribution of carbon flow through the maintenance of REDOX homeostasis (NADH/NAD^+^ ratio), regulation of metabolite concentration gradients, and modulation of enzyme activity. For instance, low-temperature conditions suppress non-enzymatic decarboxylation side reactions, whereas elevated NADH levels drive the metabolic flow toward BD accumulation (Yang et al., 2015). In previous research, it was determined that LAB and AAB are among the primary contributors to the flavor profile of naturally fermented citrus fruit vinegar (Han et al., 2024). Furthermore, studies have demonstrated that LAB and AAB are the predominant microorganisms involved in the production of high-quality grain vinegar (Chai et al., 2020). Within the fruit vinegar fermentation system, the generation of aroma compound precursors by LAB via the citrate-pyruvate metabolic axis is modulated by carbon catabolite repression and quorum sensing mechanisms (Smid and Kleerebezem, 2014). Research indicates that the expression of the ilvC-alsS operon in *Lactobacillus plantarum* is dynamically regulated by the intracellular NAD^+^/NADH ratio and pH signals (Chu et al., 2023). The citP gene in LAB encodes a citrate permease enzyme, which significantly enhances the synthetic flux of aroma compounds (Pudlik and Lolkema, 2010). It is noteworthy that there exists a competitive interaction for carbon sources between the ethanol oxidation pathway of AAB and the citric acid metabolism of LAB. This interspecies metabolic competition and cooperation can influence the efficiency of aroma compound synthesis by redistributing the metabolic flux at the pyruvate node (Zhao et al., 2024). While existing research has demonstrated an increase of over 30% in ester compounds in systems like apple cider vinegar (Feng et al., 2023), the enhancement of LAB in grain vinegar has resulted in a significant rise in acetoin content from 1827.7 mg/L to 7529.8 mg/L (Chai et al., 2020). However, the unique characteristics of citrus substrates, such as substrate compatibility issues, including the high citric acid concentration (6–8%) in citrus juice, may inhibit the activity of certain LAB strains (Nualkaekul and Charalampopoulos, 2011). Additionally, there is a competitive interaction between the ethanol oxidation process of AAB and the citric acid metabolism of LAB. This competitive interaction can be alleviated to a certain extent by controlling dissolved oxygen levels in stages (e.g., maintaining low oxygen in the early fermentation stage to promote citric acid metabolism of lactic acid bacteria, and increasing oxygen content in the later stage to facilitate ethanol oxidation of acetic acid bacteria), or by precisely regulating pH (avoiding an overly acidic environment that inhibits the activity of either type of bacteria) (Yuan et al., 2025). Additionally, a sequential inoculation approach can be adopted, where lactic acid bacteria are allowed to complete the critical stage of citric acid metabolism first before introducing acetic acid bacteria for ethanol oxidation, thereby reducing carbon source competition. Furthermore, screening for more compatible strain combinations or using genetic engineering to modify the metabolic pathways of the strains to reduce their competition for carbon sources can also help alleviate this competitive interaction, ultimately improving the synthesis efficiency of the target product. Consequently, the mechanisms for increasing acetoin through the synergistic fermentation of LAB and AAB remain unclear. Furthermore, optimizing the co-fermentation parameters, such as temperature, pH, and dissolved oxygen levels, to enhance acetoin yield warrants further investigation. In this study, the integration of strain combination screening with process coupling optimization, alongside the application of metabolomics and transcriptomics analyses, was employed to investigate the metabolic interactions between *Lactobacillus plantarum* NF2 and *Acetobacter pasteurianus* NF171. This approach aimed to elucidate the role of LAB in the biosynthesis of acetoin within citrus vinegar. The findings offer both theoretical foundations and practical frameworks for advancing high-value-added fruit vinegar products and for the microbiome engineering of food fermentation processes.

## 2 Materials and methods

### 2.1 Preparation of citrus juice

Utilizing fresh Nanfeng tangerines as the primary raw material, the process involved peeling, juicing, and removing the residue to produce citrus juice characterized by a pH of 4.16, an initial sugar concentration of 130.14 g/L, and a total acidity of 8.96 g/L. Subsequently, the sulfur dioxide (SO_2_) concentration and total sugar content of the citrus juice were adjusted to 50 mg/L and 200 g/L (Xu et al., 2022), respectively, using potassium metabisulfite (K_2_S_2_O_5_) and sucrose (Forde et al., 2011). The juice was then subjected to pasteurization at 75°C for 7 min, followed by rapid cooling to room temperature in an ice bath (Liu et al., 2018), for subsequent use.

### 2.2 Fermentation strains and culture media

*Lactobacillus plantarum* (NF2), along with the aroma-producing yeasts *Hanseniaspora guilliermondii* (Hg) and *Hanseniaspora thailandica* (Ht), were isolated and purified from naturally fermented citrus wine. *Acetobacter pasteurianus* (NF171) was isolated and identified from naturally fermented citrus vinegar in previous laboratory research. *Saccharomyces cerevisiae* (Sc) was procured from *Saccharomyces Cerevisiae* Co., Ltd. *Lactobacillus plantarum* was cultured using MRS broth medium. All yeasts and *Acetobacter pasteurianus* were cultured using YPD medium. Sc is a commercial *Saccharomyces cerevisiae*, while both Hg and Ht are non-Saccharomyces yeasts preserved in the laboratory. *Saccharomyces cerevisiae* exhibits strong fermentation performance, while non-Saccharomyces yeasts primarily enhance the flavor of food products (Vicente et al., 2022). All strains required for the experiment were preserved in the laboratory refrigerator at −80°C.

### 2.3 Fermentation process

The yeast cultivation method was employed as previously described, with minor modifications (Xu et al., 2022). Four distinct citrus wine fermentation combinations were established utilizing Sc, along with aroma-producing yeasts Hg and Ht. These combinations were as follows: (A) pure fermentation with Sc; (B) sequential fermentation where Hg was initially introduced into the juice, followed by inoculation with Sc after 24 h; (C) mixed fermentation involving both Hg and Ht; and (D) co-inoculation of Hg and Sc at a 1:1 ratio. Each yeast strain was introduced at a concentration of 10^7^ CFU/mL. Additionally, *Lactobacillus plantarum* NF2 was co-inoculated at a concentration of 10^7^ CFU/mL during three distinct stages of fermentation: the early stage (S) at the onset of fermentation, the middle stage (M) when the residual sugar content decreased to 80–120 g/L, and the late stage (E) when the residual sugar content fell below 30 g/L as Table 3 showed. Each experimental condition was conducted in triplicate. The fermentation process was carried out at a temperature of 28°C, with static incubation over a period of 5 days. The conclusion of the citrus wine fermentation was determined when the residual sugar content dropped below 5 g/L and the lactic acid concentration remained stable for two consecutive days. After the fermentation of the citrus wine is completed, centrifuge at 4,200 rpm for 30 min and take the supernatant for later use.

**Table 3:** Fermentation and combination of fruit wine.

Series	Control	Early stage of alcoholic fermentation (S)	Mid-stage of alcoholic fermentation (M)	Late stage of alcoholic fermentation (E)
A	Sc (A)	A-NF2 (AS)	A-NF2 (AM)	A-NF2 (AE)
B	Hg-10-Sc (B)	B-NF2 (BS)	B-NF2 (BM)	B-NF2 (BE)
C	Hg: Ht-1:1 (C)	C-NF2 (CS)	C-NF2 (CM)	C-NF2 (CE)
D	Sc: Hg-1:1 (D)	D-NF2 (DS)	D-NF2 (DM)	D-NF2 (DE)

Using citrus wine supplemented with 10 g/L glucose as the substrate, it was pasteurized at 75°C for 25 min and then rapidly cooled to room temperature for later use. The control group comprised four groups of citrus wines that were not inoculated with LAB during the fermentation process and were only inoculated with *Acetobacter pasteurianus* NF171 for citrus vinegar fermentation. The experimental group consisted of citrus wines inoculated with LAB during fermentation. *Lactobacillus plantarum* NF2 and *Acetobacter pasteurianus* NF171 were inoculated at three stages corresponding to acetic acid fermentation to co-ferment the citrus vinegar. Both *Lactobacillus plantarum* NF2 and *Acetobacter pasteurianus* NF171 were inoculated at a concentration of 10^7^ CFU/mL. In the experimental group, *Lactobacillus plantarum* NF2 was introduced at a concentration of 10^7^ CFU/mL during the early stage of acetic acid fermentation (S, simultaneously inoculated with *Acetobacter pasteurianus* NF171), (M, acetic acid ≥10 g/L), and (E, acetic acid ≥25 g/L) based on the fermentation progress of the citrus vinegar. The fermentation conditions included a stirring speed of 160 rpm, a temperature of 31°C, and a fermentation period of 9 days. When the concentrations of acetoin and acetic acid in the system ceased to increase, the citrus vinegar fermentation was deemed complete. Subsequently, the citrus vinegar was centrifuged at 8,000 rpm for 10 min to remove bacterial residues, resulting in citrus vinegar. The specific fermentation combinations are detailed in Table 4.

**Table 4:** Combination of fruit vinegar fermentation.

Series	Control	Early stage of vinegar fermentation (S)	Mid-stage of vinegar fermentation (M)	Late stage of vinegar fermentation (E)
A	Sc (AF)	A-NF2 (ASS)	A-NF2 (AMM)	A-NF2 (AEE)
B	Hg-10-Sc (BF)	B-NF2 (BSS)	B-NF2 (BMM)	B-NF2 (BEE)
C	Hg: Ht-1:1 (CF)	C-NF2 (CSS)	C-NF2 (CMM)	C-NF2 (CEE)
D	Sc: Hg-1:1 (DF)	D-NF2 (DSS)	D-NF2 (DMM)	D-NF2 (DEE)

### 2.4 Chemical composition analysis of citrus vinegar

The components of citrus vinegar were measured as previously described (Yu et al., 2019; Qiu et al., 2021; Xu et al., 2022). In accordance with the National Food Safety Standard GB 12456-2021, the total acidity in the food sample was quantified using sodium hydroxide titration, while the ethanol content was determined via gas chromatography coupled with a flame ionization detector (GC-FID). The concentrations of nine organic acids, including vitamin C, lactic acid, oxalic acid, malic acid, fumaric acid, maleic acid, citric acid, succinic acid, and acetic acid, were measured using high-performance liquid chromatography (HPLC). The organic acids present in citrus wine and citrus vinegar were both qualitatively and quantitatively assessed using the external standard method. Phenolic acids were similarly analyzed by HPLC at various wavelengths. The detection of 10 flavonoid types in the samples was conducted using HPLC, specifically targeting sinocatrin, naringin, hesperidin, neohesperidin, gerberin, and naringin at 283 nm, as well as sweet orange flavonoids and nobiletin at 330 nm. Acetoin was also qualitatively and quantitatively analyzed using HPLC, with certain modifications applied based on the technical specifications of the instrument and chromatographic column.

### 2.5 Optimization of acetoin production

#### 2.5.1 Effects of inoculation amount of fermentation strains on acetoin production

Connect *Acetobacter pasteurianus* NF171 and *Lactobacillus plantarum* NF2 in a 1:1 ratio. The inoculation amounts were adjusted to 1, 3, 5, 7, 9, and 11% (5% inoculation amount corresponds to 10^7^ CFU/mL). The fermentation temperature was 31°C, the rotational speed was 160 rpm, and the fermentation period was 9 days. Three parallel groups were set for each combination.

#### 2.5.2 Effects of rotational speed on acetoin production

Connect *Acetobacter pasteurianus* NF171 and *Lactobacillus plantarum* NF2 in a 1:1 ratio. The inoculation amount of each strain was 3%. The fermentation temperature was 31°C, and the rotational speeds were set at 0, 40, 80, 120, 160, and 200 rpm. The culture was carried out for 9 days, and three parallel settings were set for each combination.

#### 2.5.3 Effects of fermentation temperatures on acetoin production

Connect *Acetobacter pasteurianus* NF171 and *Lactobacillus plantarum* NF2 in a 1:1 ratio. The inoculation amount of each strain was 3%, the rotational speed was 120 rpm, the fermentation temperatures were set at 28, 31, 33, 35, 37 and 40°C, and the culture was carried out for 9 days. Three parallel settings were set for each combination.

#### 2.5.4 Effects of sugar supplementation on acetoin production

Connect *Acetobacter pasteurianus* NF171 and *Lactobacillus plantarum* NF2 in a 1:1 ratio. The inoculation amount of each strain was 3%, the rotational speed was 120 rpm, the fermentation temperature was set at 33°C, the glucose of the citrus wine was adjusted to 10, 20, 30, 40, and 60 g/L, and the fermentation was carried out for 9 days. Three parallel settings were set for each combination.

### 2.6 Transcriptome analysis

Minor modifications were implemented based on the previously described method (Cheng et al., 2025). During the fermentation of citrus wine, samples were collected on the 2nd and 5th days. The fermentation process utilized single strains of Hg and Ht, as well as a mixed fermentation of both strains. Each treatment group included three parallel samples, with each sample having a volume of 15 mL. RNA extraction from the fermentation process was performed using the RNAprep Pure Cell/Bacterial Kit (Tiangen Biotechnology, Beijing, China). The size distribution of the fragments was determined using the Qsep400 High-Throughput Analysis System, and the effective concentration of the libraries was accurately measured by quantitative PCR (qPCR). Sequencing was conducted on the Illumina NovaSeq6000 platform. Data processing was carried out in accordance with the previously described procedure (Deng et al., 2024). Valid reads were aligned to a reference genome sequence, and only perfectly matched sequences or those with a single mismatch were analyzed and annotated. Functional annotation and metabolic pathway enrichment analysis of differentially expressed genes were performed based on the Kyoto Encyclopedia of Genes and Genomes (KEGG) database.^1^

### 2.7 Statistical analysis

The data, including error bars, are shown as the mean ± SD of triplicate, values with different Roman letters in the same row indicating significant differences at *p* < 0.05 (Duncan’s test).

## 3 Results and discussion

### 3.1 Mid-stage inoculation of *Lactobacillus plantarum* NF2 significantly enhances acetoin production

**Figure 1:**
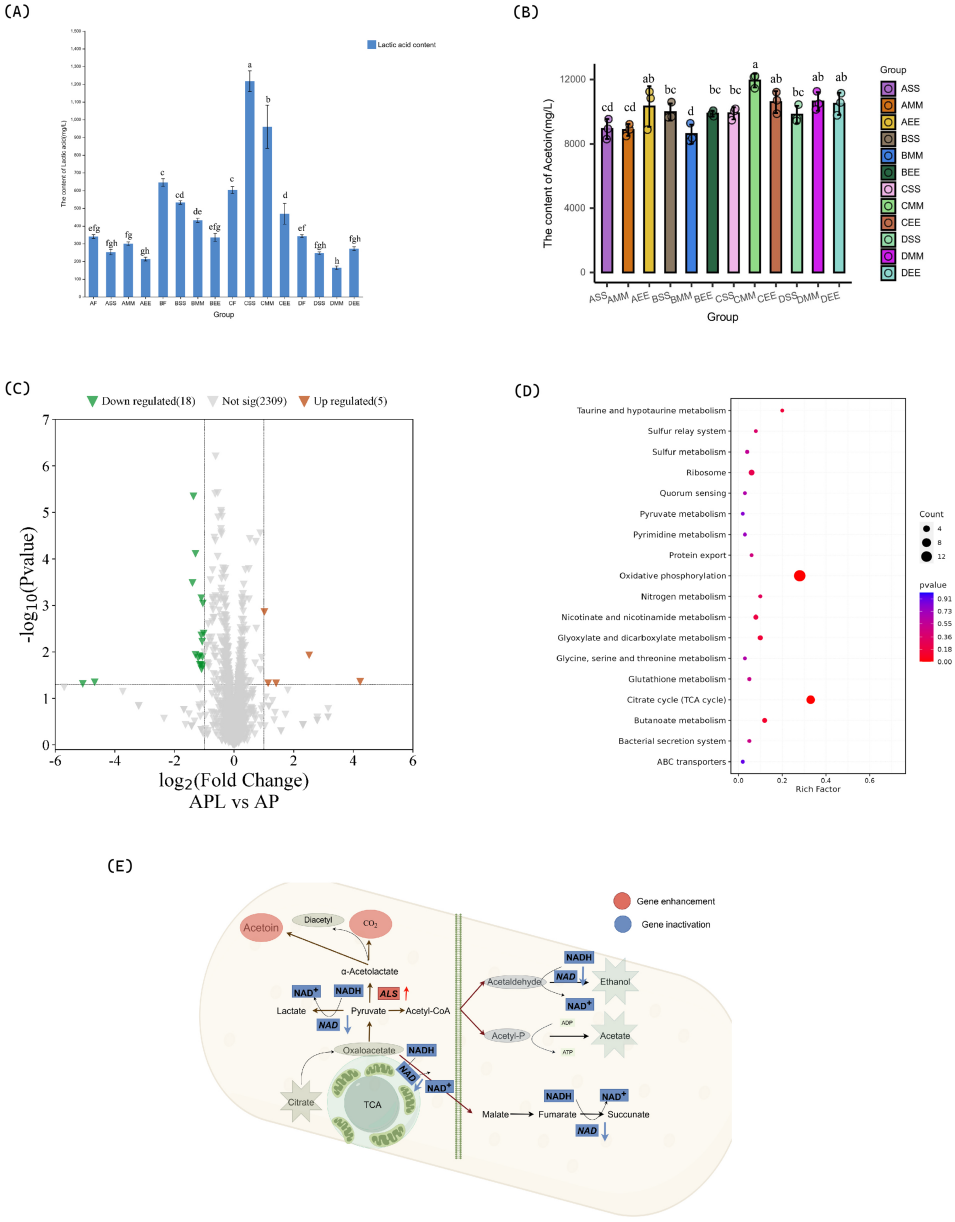
Changes in substances and pathways during citrus vinegar fermentation and related mechanisms. **(A)** Changes of lactic acid during citrus vinegar fermentation with different combinations. **(B)** Changes of acetoin during citrus vinegar fermentation with different microbial combinations. **(C)** Volcano plot of genes. **(D)** KEGG metabolic pathways. **(E)** Mechanism of action during citrus vinegar fermentation. Data shown are the mean ± SD of triplicate, values with different Roman letters in the same row indicating significant differences at *p* < 0.05 (Duncan’s test).

Lactic acid functions not only as a metabolic byproduct in microbial fermentation but also plays a crucial role in enhancing acetoin formation by modulating pH levels and metabolic pathway activity (Kuhn et al., 2025). Within the A series combination, the lactic acid content in the citrus wine substrate follows the order: AS > AM > AE > A. During the initial phase of acetic acid fermentation, acetoin content increased rapidly, while the reduction in lactic acid content was significantly greater than the amount of acetoin produced. This suggests that lactic acid is utilized not only in the synthesis of acetoin but also by *Acetobacter pasteurianus* NF171 for growth and proliferation. The acetoin content in ASS reached its peak at 963.74 ± 10.92 mg/L, which was delayed inoculation of *Lactobacillus plantarum* NF2 led to reduced acetoin due to acetic acid inhibition. This finding indicates that earlier inoculation of *Lactobacillus plantarum* NF2 is advantageous for the accumulation of acetoin in series A citrus vinegar. In the later stages of acetic acid fermentation of AEE, the inoculation timing of *Lactobacillus plantarum* NF2 was suboptimal, occurring too late. This delay resulted in an excessive concentration of acetic acid, which subsequently inhibited the growth and metabolic activity of *Lactobacillus plantarum* NF2. The trend in lactic acid levels mirrored that of compound A, with a continuous decline and no subsequent increase in lactic acid content. Similarly, the concentration of acetoin followed a comparable pattern. Additionally, the accumulation of acetoin in each experimental combination peaked on the fifth day, after which a decline occurred (Figures 1A,B).

During the fermentation process of the B series combined citrus vinegar, the lactic acid consumption rate was moderate. However, when *Lactobacillus plantarum* NF2 was inoculated at various stages of acetic acid fermentation, there was no increase in lactic acid content in the citrus vinegar. This may be attributed to the lactic acid production rate by *Lactobacillus plantarum* NF2 being lower than the consumption rate by *Acetobacter pasteurianus* NF171. Notably, the accumulation of acetoin in the BSS combination was the highest at 805.25 mg/L, followed by the BMM combination at 673.45 mg/L. The BEE and BF combinations exhibited acetoin concentrations of 281.36 mg/L and 386.07 mg/L, respectively (Figures 1A,B).

Within the C series of citrus vinegars, the lactic acid concentrations in citrus wines CS and CM were measured at 7.1 g/L and 11.4 g/L, respectively (Figures 1A,B). In contrast, the acetoin yields in CSS and CMM citrus vinegars were recorded at 1137.92 ± 68.48 mg/L and 1616.95 ± 54.67 mg/L, respectively. Furthermore, when CSS and CMM were subjected to acetic acid fermentation with inoculation by *Lactobacillus plantarum* NF2, a metabolic process ensued that temporarily elevated the lactic acid content in the citrus vinegar. This observation aligns with the metabolic trend results observed for *Lactobacillus plantarum* NF2 in series A. In comparison, the acetoin content in the D series citrus vinegar was determined to be lower than in series A, B, and C. This discrepancy may be attributed to the relatively low initial lactic acid content and the relatively high acetic acid content in the citrus wine substrate of the D series. Consequently, the accelerated acid production rate in the D series appears to impede the growth and metabolism of *Lactobacillus plantarum* NF2, limiting the lactic acid available to *Acetobacter pasteurianus* NF171 and resulting in a reduced accumulation of acetoin. This could be because lactic acid can be utilized by *Acetobacter pasteurianus* NF171 and metabolically converted into pyruvate, which is a key precursor for acetoin synthesis. The accumulation of lactic acid provides an adequate carbon source for pyruvate, thereby promoting the carbon flow in the acetoin synthesis pathway (Cui et al., 2023).

The high-yield acetoin fermentation combination CMM was evaluated using *Lactobacillus plantarum* NF2. The acetoin content in citrus vinegar produced without *Lactobacillus plantarum* NF2 inoculation was significantly lower compared to that produced with *Lactobacillus plantarum* NF2 co-fermentation introduced during the early and middle stages. An increase in lactic acid content in citrus wine correlates with a higher accumulation of acetoin in citrus vinegar. Notably, the acetoin content in citrus vinegar fermented using the CMM combination was the highest (1616.95 ± 54.67 mg/L). This level was substantially greater than the acetoin content documented in hawthorn vinegar (698.35 ± 6.67 mg/L) (Özdemir et al., 2021), and in apple vinegar co-fermented by LAB and AAB (770 mg/L) (Zhang et al., 2022). The strategic inoculation of *Lactobacillus plantarum* NF2 during alcoholic fermentation enhances lactic acid production and facilitates increased accumulation of acetoin in citrus vinegar.

### 3.2 “Energy metabolism regulation-carbon flow directional guidance” drives acetoin biosynthesis

To further investigate the mechanism by which *Lactobacillus plantarum* NF2 enhances the metabolism of *Acetobacter pasteurianus* NF171 and augments the synthesis of acetoin in citrus vinegar, the gene transcription involved in the synergistic fermentation of *Lactobacillus plantarum* NF2 and *Acetobacter pasteurianus* NF171 was analyzed using transcriptomic techniques. In the *Lactobacillus plantarum* NF2-supplemented fermented citrus vinegar group (APL), a total of 2,332 genes were expressed, with 23 DEGs identified, comprising 5 up-regulated and 18 down-regulated genes. The results of the KEGG enrichment analysis of DEGs are presented in Figure 1C. The enrichment occurred via several metabolic pathways, including oxidative phosphorylation, the citrate cycle (TCA cycle), butanoate metabolism, glyoxylate and dicarboxylate metabolism, taurine and hypotaurine metabolism, nicotinate and nicotinamide metabolism, ribosome function, nitrogen metabolism, the sulfur relay system, protein export, the bacterial secretion system, glutathione metabolism, sulfur metabolism, glycine, serine, and threonine metabolism, quorum sensing, pyrimidine metabolism, pyruvate metabolism, and ABC transporters.

Through KEGG and GO enrichment analyses of significantly differentially expressed genes in Figure 1D, alterations were identified in the pathways associated with NADH dehydrogenase complex assembly and pyruvate metabolism. These findings suggest a metabolic reprogramming strategy in mixed bacterial fermentation, with a core mechanism centered on “energy metabolism regulation” and “carbon flow directional guidance” (Figure 1E). The down-regulation of the NADH metabolic pathway results in NADH accumulation and subsequent inhibition of the respiratory chain. Metabolomic analysis indicates a declining trend in the levels of citric acid and succinic acid in APL citrus vinegar, suggesting an inhibition of the metabolic pathway converting citric acid from oxaloacetic acid to succinic acid. Consequently, citric acid metabolism shifts toward the conversion from oxaloacetic acid to pyruvic acid. Additionally, ethanol in citrus vinegar is converted to acetic acid via the acetyl-CoA cycle under the oxidation of *Acetobacter pasteurianus* NF171, which partially impedes the conversion of pyruvate to acetic acid through the tricarboxylic acid cycle. Furthermore, during the fermentation process of citrus vinegar, *Lactobacillus plantarum* NF2 produces lactic acid and releases it into the citrus vinegar, which subsequently enhances the accumulation of pyruvate within *Acetobacter pasteurianus* NF171. When lactic acid is taken up by microorganisms (such as *Acetobacter pasteurianus* NF171) as a carbon source, it undergoes an oxidation reaction catalyzed by enzymes like lactate dehydrogenase to generate pyruvate. This conversion process directly increases the intracellular pyruvate pool. Meanwhile, the continuous supply of lactic acid provides a stable substrate source for this metabolic pathway, preventing excessive consumption of pyruvate due to its involvement in other metabolic routes (such as entry into the tricarboxylic acid cycle). This thus promotes the accumulation of pyruvate within the cell, supplying sufficient precursors for the subsequent synthesis of compounds like acetoin (Li et al., 2017). Notably, the ALS gene, which encodes the first rate-limiting enzyme in the acetoin synthesis pathway, was significantly upregulated. This upregulation directly facilitates the conversion of pyruvate to α-acetyllactic acid, thereby providing additional precursors for acetoin synthesis, increasing the carbon influx, and promoting acetoin production in citrus vinegar. It is crucial to maintain carbon flow stability during the ethylene stage through regulation. This finding contrasts with previous studies, where in the mixed bacterial fermentation of rice and apple vinegar, the ALS gene was also upregulated, but pyruvic acid did not accumulate. Pyruvic acid primarily entered the TCA cycle to produce acetic acid (Akasaka et al., 2013; Li et al., 2023). The metabolism of citric acid in blueberry vinegar remained uninhibited, with the majority of oxaloacetic acid being converted into succinic acid, thereby leading to a deficiency in pyruvate precursors (Benziman et al., 1978; Jiang et al., 2022). Similarly, the activity of NADH dehydrogenase in wine vinegar was not impeded, allowing the electron transport chain to continuously consume reducing power (Smyth and Orsi, 1989; Qi et al., 2014; Wei et al., 2024), all of which led to the low synthesis of acetoin. This study offers insights into enhancing specific metabolites in mixed bacterial fermented citrus vinegar through metabolic reprogramming strategies.

### 3.3 Mixed bacterial fermentation enhances the flavor substances of citrus vinegar

**Figure 2:**
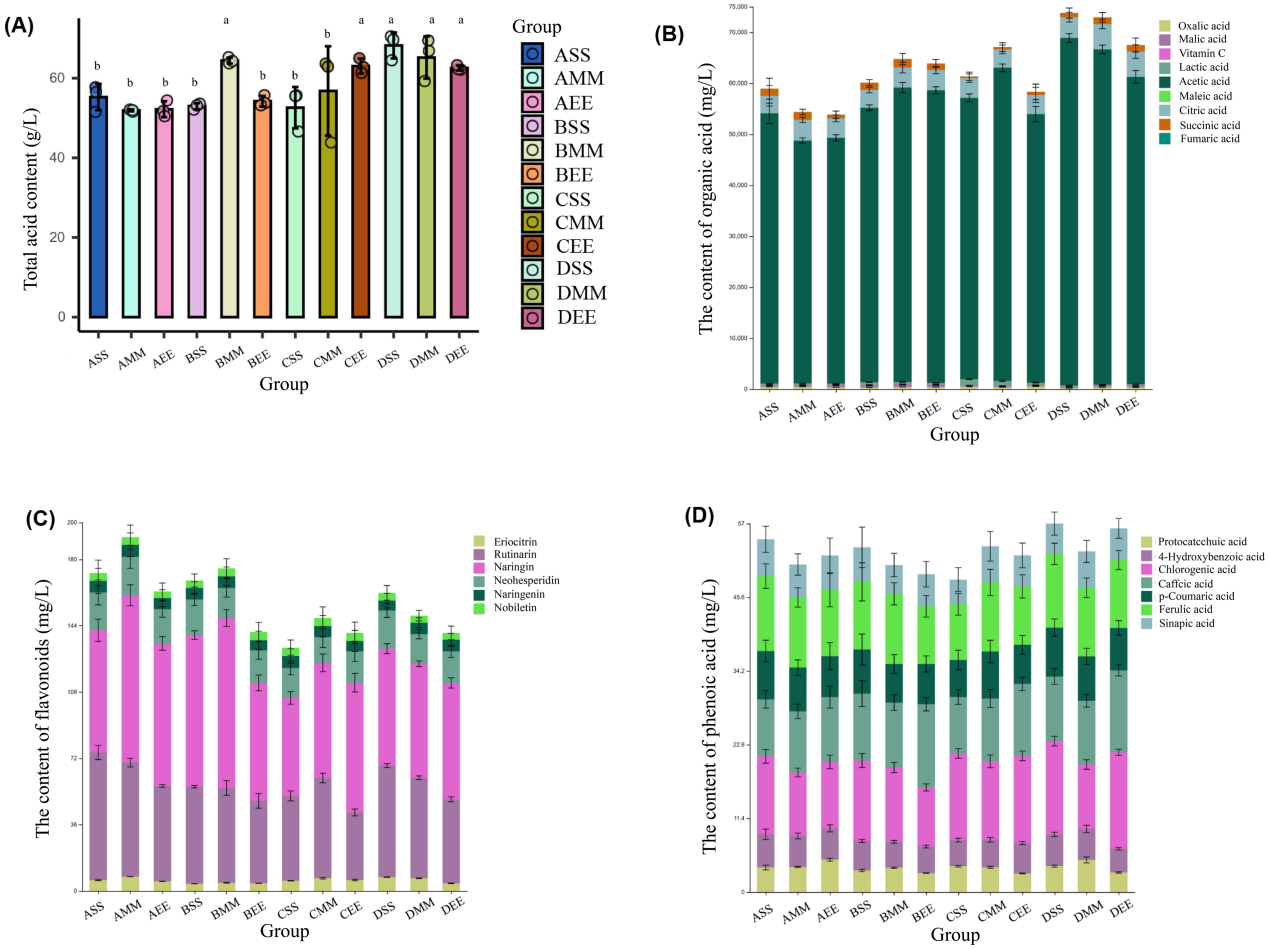
Changes in flavor compounds during citrus vinegar fermentation with different microbial combinations. **(A)** Changes in total acid content during citrus vinegar fermentation with different microbial combinations. **(B)** Changes in organic acids during citrus vinegar fermentation with different microbial combinations. **(C)** Changes in flavone during citrus vinegar fermentation with different microbial combinations. **(D)** Changes in phenolic acids during citrus vinegar fermentation with different microbial combinations. Data shown are the mean ± SD of triplicate, values with different Roman letters in the same row indicating significant differences at *p* < 0.05 (Duncan’s test).

Following acetic acid fermentation, with reference to GB 12456-2021 National Food Safety Standard for the determination of total acids in food, the total acid concentration in each citrus vinegar formulation surpassed 40 g/L, thereby complying with the national standards for citrus vinegar products (Figure 2A). The total acid content in citrus vinegar co-fermented with *Lactobacillus plantarum* NF2 and *Acetobacter pasteurianus* NF171 was higher than that in vinegar fermented solely with *Acetobacter pasteurianus* NF171 (Table 1). The polyphenolic compounds in citrus vinegar predominantly consist of phenolic acids, such as chlorogenic acid and ferulic acid, and flavonoids, including neohesperidin and rutinarin. The presence and spatial–temporal distribution of these compounds are significantly modulated by *Lactobacillus plantarum* NF2 (Table 2). In the *Lactobacillus plantarum* NF2 intervention group, there was a general increase in the total phenolic acid content, with the DSS group exhibiting particularly notable enhancements: chlorogenic acid and ferulic acid concentrations rose by 62 and 89%, respectively, compared to the DF group, reaching levels of 14.42 mg/L and 11.40 mg/L. The flavonol lineage exhibits phased regulatory characteristics (Figures 2B–D). Notably, the accumulation of rutinarin in the early and mid-stage inoculation group during acetic acid fermentation, particularly peaking in the AMM group, was elevated compared to the control group (Table 2). This increase may be attributed to the glycoside hydrolase activity of *Lactobacillus plantarum* NF2. In the NF2 intervention groups, particularly the AMM group, the accumulated amount of rutin was significantly higher than that in the control group. This phenomenon *might potentially be linked* to the *putative* glycoside hydrolase activity of NF2, though such activity has not yet been directly verified through experiments. Glycoside hydrolases are generally known to be capable of catalyzing the cleavage of glycosidic bonds in flavonoid glycosides (Kotik et al., 2021). It is therefore *speculated that* NF2 *could* promote the release and accumulation of flavonoids such as rutin through a similar mechanism. However, this proposed mechanism *would still need* further confirmation through subsequent specific detection of enzyme activity, such as *in vitro* enzyme activity experiments or related gene expression analysis. Furthermore, naringin was specifically enriched in the ASS group, reaching a concentration of 69.34 mg/L. The enrichment of polyphenols in citrus vinegar demonstrated in this study exceeds the levels previously reported in the literature (Han et al., 2024). These results indicate that the spatio-temporal intervention of *Lactobacillus plantarum* NF2 enhances the active substances of citrus vinegar.

### 3.4 Process optimization has significantly increased the content of acetoin in citrus vinegar

The synthesis and accumulation of acetoin result from the synergistic metabolism of AAB and LAB, necessitating the optimization of parameters in a two-stage fermentation process. Based on previous experimental findings, it has been determined that both the production and accumulation of acetoin occur during the acetic acid fermentation stage. Consequently, our research aimed at enhancing the acetoin content in citrus vinegar was concentrated on this stage. Lactic acid serves as a crucial carbon source for acetoin synthesis, with the CM combination citrus wine exhibiting the highest lactic acid content. The acetoin content in citrus vinegar, derived from acetic acid fermentation with the introduction of *Lactobacillus plantarum* NF2 during the intermediate stage, was found to be the highest when using this substrate. Therefore, we selected the CMM fermentation method as the focus of our study. To optimize the fermentation conditions for high-yield acetoin production, a single-factor optimization experiment was conducted, using the acetoin content in citrus vinegar as the primary evaluation index, while also considering the yield of acetic acid and the lactic acid content in the citrus vinegar.

#### 3.4.1 The influence of inoculation amount on the accumulation of acetoin in citrus vinegar

**Figure 3:**
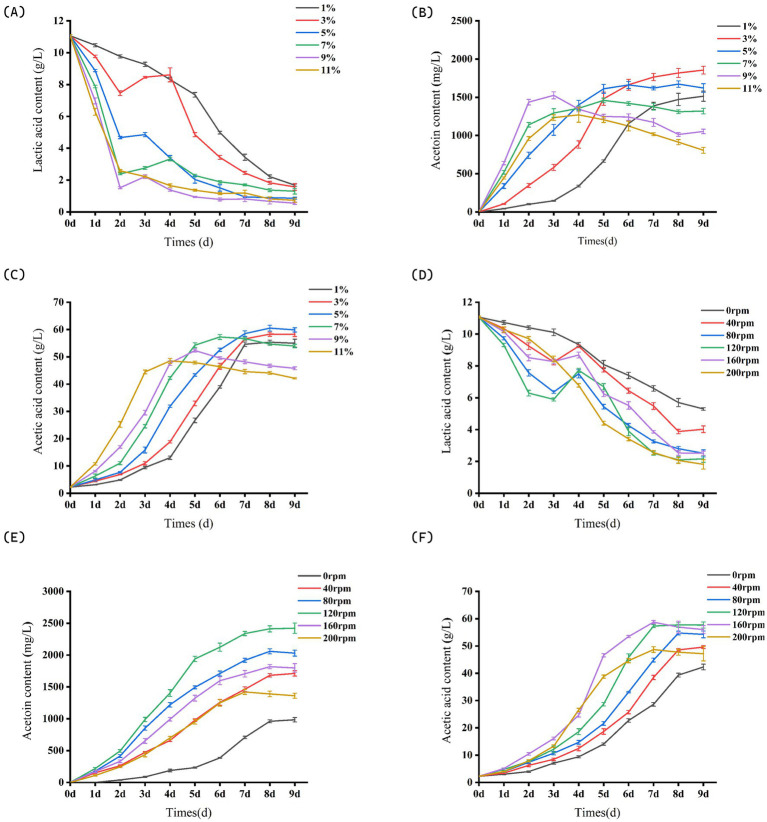
Partial parameters for acetoin process optimization. **(A)** Effects of different inoculation amounts on lactic acid content in fruit vinegar. **(B)** Effects of different inoculation amounts on acetoin content in fruit vinegar. **(C)** Effects of different inoculation amounts on acetic acid content in fruit vinegar. **(D)** Effects of different rotation speeds on lactic acid content in fruit vinegar. **(E)** Effects of different rotation speeds on acetoin content in fruit vinegar. **(F)** Effects of different rotation speeds on acetic acid content in fruit vinegar. Data shown are the mean ± SD of triplicate.

Under inoculation conditions of 9 and 11%, the acetic acid content in citrus vinegar peaked on the fourth and fifth days (Figure 3C), respectively. Additionally, the acetoin content in citrus vinegar with a 9% inoculation reached its maximum on the third day (Figure 3B), thereby shortening the fermentation cycle. However, during the later stages of fermentation, the concentrations of both acetic acid and acetoin declined. This decline may be attributed to an excessive concentration of citrus vinegar bacteria, which likely led to the over-oxidation of acetic acid, acetoin was consumed and metabolized as a reserve energy source. “over-oxidation” refers to a metabolic state where *Acetobacter pasteurianus* NF171, under conditions such as excessively high inoculation levels (e.g., 9% or 11% as observed) or prolonged fermentation, exhibits non-specific oxidative metabolism beyond the normal scope of target product synthesis. Biologically, this arises from AAB’s strong oxidative capacity: while NF171 primarily oxidizes ethanol to acetic acid under optimal conditions, high bacterial density or carbon source limitation can trigger it to extend oxidative pathways to utilize non-preferred substrates, including intended accumulated metabolites like acetoin. This reduces acetoin through two key mechanisms: first, acetoin, as a carbon-containing intermediate, is directly oxidized via secondary metabolic pathways—potentially broken down into simpler compounds (e.g., acetyl-CoA) that enter the TCA cycle and are ultimately converted to CO₂ and water, as seen in the late-stage decline of acetoin under high-inoculum fermentation (Figure 3B); second, over-oxidation consumes reducing equivalents like NADH, altering the NADH/NAD^+^ ratio, which disrupts the redox homeostasis critical for acetoin synthesis (as transcriptome analysis revealed downregulated NADH flux promotes acetoin accumulation), thereby shifting metabolic flux away from acetoin biosynthesis and indirectly inhibiting its production. Thus, over-oxidation reflects a metabolic imbalance in NF171, directly degrading acetoin and disrupting its synthetic environment to reduce accumulation.

When the inoculation level was below 9%, the final concentration of acetic acid in the citrus vinegar fermentation system for each inoculation level ranged between 55 and 60 g/L, with no significant differences observed. The highest accumulation of acetoin occurred at an inoculation level of 3% (1855.21 ± 49.58 mg/L), and the residual lactic acid content was also relatively high (1.57 ± 0.21 g/L) (Figure 3A). From the above, it can be known that the content of acetoin and lactic acid in citrus vinegar produced with an inoculation amount of 3% is superior to that of other inoculation amounts. To facilitate the further optimization of fermentation conditions, a 3% inoculum concentration has been identified as the appropriate choice.

#### 3.4.2 The influence of oxygen supply intensity on the accumulation of acetoin in citrus vinegar

In the CMM fermentation system, *Lactobacillus plantarum* NF2 is classified as a facultative anaerobic bacterium, while *Acetobacter pasteurianus* NF171 is categorized as an aerobic bacterium. The level of oxygen supply exerts a significant influence on the fermentation process of citrus vinegar. *Lactobacillus plantarum* NF2 is optimized for the metabolic production of lactic acid under hypoxic or low-oxygen conditions, whereas *Acetobacter pasteurianus* NF171’s metabolic activity necessitates a substantial oxygen supply. Consequently, it is imperative to determine an optimal oxygen concentration that facilitates acetoin accumulation in the citrus vinegar fermentation system. As fermentation progresses, acetoin accumulates rapidly across various oxygen supply intensities, with the exception of the 0 rpm group. Under the condition of 120 rpm, acetoin accumulation reaches its peak (2421.95 ± 80.68 mg/L) (Figure 3E). When the shake flask rate falls below 120 rpm (Figure 3D), the acetoin accumulation decreases, indicating that *Acetobacter pasteurianus* NF171 requires a specific oxygen intensity for effective metabolism. As the shake flask rate increases, the maximum accumulation of acetoin is achieved. The high rotational speed generates shear forces that adversely affect *Acetobacter pasteurianus* NF171, thereby inhibiting acetoin production and lactic acid consumption within the system (Qian et al., 2023). Furthermore, the excessive oxygen dissolution resulting from elevated rotational speeds also impedes the lactic acid production efficacy of *Lactobacillus plantarum* NF2 (Figure 3F). Relevant studies have shown that rotational speed is associated with damage to bacteria: centrifugation can alter bacterial cell surface properties and internal structures (including DNA). The high shear forces generated by high rotational speeds during centrifugation can cause damage to bacterial cell surfaces, affecting their surface characteristics and related experimental results. This bears similarities to the damage suffered by acetic acid bacteria under high rotational speeds (Peterson et al., 2011). When the shake flask rate exceeds 160 rpm, the maximum acetic acid yield is significantly lower compared to other groups.

#### 3.4.3 The influence of fermentation temperature on the yield of acetoin in citrus vinegar

**Figure 4:**
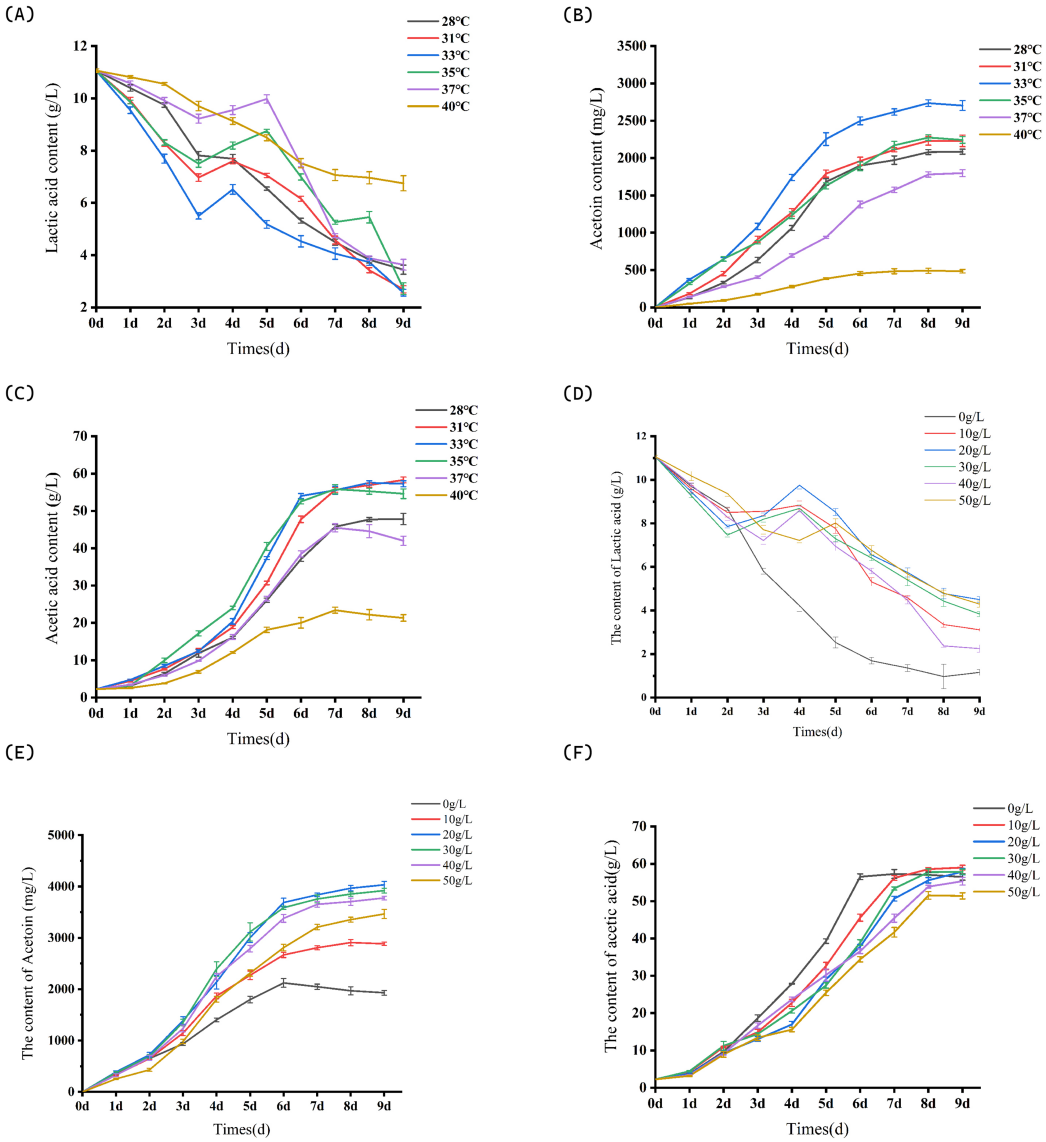
Partial parameters for acetoin process optimization. **(A)** Effects of different fermentation temperatures on lactic acid content in fruit vinegar. **(B)** Effects of different fermentation temperatures on acetoin content in fruit vinegar. **(C)** Effects of different fermentation temperatures on acetic acid content in fruit vinegar. **(D)** Effects of different glucose concentrations on lactic acid content in fruit vinegar. **(E)** Effects of different glucose concentrations on acetoin content in fruit vinegar. **(F)** Effects of different glucose concentrations on acetic acid content in fruit vinegar. Data shown are the mean ± SD of triplicate, values with different Roman letters in the same row indicating significant differences at *p* < 0.05 (Duncan’s test) in Figure 4A.

Prior to reaching 33°C, an increase in system temperature results in a continuous acceleration of both the generation rate of acetoin and the consumption rate of lactic acid. However, when the fermentation temperature surpasses 33°C, the consumption rate of lactic acid within the citrus vinegar fermentation system decelerates. At 40°C, the accumulation of acetoin is minimal at this temperature (Figure 4A), measuring 484.00 ± 23.59 mg/L (Figure 4B), which underscores the substantial impact of temperature on acetoin production. The total acetic acid content in systems maintained at 31°C, 33°C, and 35°C is comparable (Figure 4C), while the highest acetoin yield is achieved at 33°C, reaching 2703.12 ± 65.96 mg/L.

#### 3.4.4 The influence of glucose concentration on the yield of acetoin citrus vinegar

When the glucose supplementation level was below 20 g/L, the accumulation of acetoin in citrus vinegar was lower compared to other glucose supplementation systems. Notably, the 20 g/L glucose supplementation system exhibited the highest acetoin content, measuring 4033.72 ± 64.48 mg/L (Figure 4E). Glucose supplementation has been shown to enhance lactic acid production during the fermentation process of citrus vinegar in Figure 4D. Within a specific range, the yield of lactic acid is directly proportional to the level of sugar supplementation. However, when sugar supplementation exceeds 40 g/L, both the generation and consumption rates of lactic acid decrease, leading to a reduction in acetoin accumulation. As illustrated in Figure 4E, with the exception of the 50 g/L system, the accumulation of acetic acid in citrus vinegar under varying sugar supplementation conditions remains relatively consistent, ranging from 56 to 60 g/L. The excessively high sugar concentration may induce stress in the *Acetobacter pasteurianus* NF171 strain due to the high-sugar environment, thereby diminishing its metabolic capacity for acetic acid production. In the context of this study, sugar stress inhibits *Acetobacter pasteurianus* NF171 through a combination of interconnected mechanisms, as supported by experimental observations. When glucose supplementation exceeds 40 g/L, the hyperosmotic environment created by high sugar concentrations causes osmotic stress: water is drawn out of NF171 cells, disrupting cellular structures and membrane integrity, which impairs critical functions such as nutrient uptake and metabolite secretion. This osmotic damage directly reduces the bacterium’s metabolic activity, as evidenced by decreased acetic acid production under excessive sugar conditions (≥50 g/L) in Figure 4F.

Metabolically, excessive glucose overwhelms NF171’s metabolic pathways, skewing carbon flux distribution. The study shows that high sugar levels reduce lactic acid consumption rates, limiting the availability of pyruvate—a key precursor for acetoin synthesis. This metabolic imbalance diverts resources away from primary pathways (e.g., acetic acid and acetoin production) toward stress responses. NF171 likely expends additional energy on synthesizing osmoprotectants or repairing damaged proteins, further diverting resources from productive metabolism.

Collectively, these effects—osmotic disruption, metabolic flux misregulation, and energy allocation to stress mitigation—suppress NF171’s growth, enzymatic activity, and ability to synthesize target metabolites, resulting in reduced fermentation efficiency under sugar stress.

In comparison to other citrus vinegar products, the accumulation of acetoin in this process (4033.72 mg/L) was markedly higher than the values reported in the literature. For instance, the acetoin content in traditional apple vinegar typically remains below 1,000 mg/L (Zhang et al., 2022), while the maximum concentration attained in jujube vinegar following secondary fermentation was only 2,150 mg/L (Jo et al., 2015). These findings suggest that the regulation of the synergistic metabolism between *Lactobacillus plantarum* NF2 and *Acetobacter pasteurianus* NF171, in conjunction with a precise sugar supplementation strategy, has facilitated the efficient synthesis of acetoin. This approach offers a novel pathway for the development of flavor-enhanced citrus vinegar.

## 4 Conclusions and prospects

In this study, a microbiome engineering strategy integrating strain adaptation and process optimization was applied to significantly enhance acetoin production during citrus vinegar fermentation—from less than 500 mg/L to 4033.72 mg/L—approaching the levels typically documented in traditional rice vinegar (Chai et al., 2020). The co-fermentation system composed of *Lactobacillus plantarum* NF2 and *Acetobacter pasteurianus* NF171 promoted acetoin accumulation through the “lactic acid–pyruvate–acetoin” metabolic route. Lactic acid, produced by *Lactobacillus plantarum* NF2 via sugar metabolism, served as both a carbon source for *Acetobacter pasteurianus* NF171 and a pH-lowering agent that activated pyruvate metabolism. This led to significant upregulation of the *als* gene, enhancing the flux of pyruvate toward acetoin biosynthesis. Transcriptomic analysis further revealed persistent downregulation of genes involved in the TCA cycle, suggesting that overexpression of the *citP* gene through genetic engineering may enhance acetoin yield in future studies. Notably, the current work was limited to single-factor optimization. Subsequent studies should employ orthogonal experimental designs to validate and expand these findings. Additionally, late-stage fermentation exhibited challenges such as insufficient lactic acid supply and product degradation. Such issues can be eased with strategies like two-stage pH control or *in situ* lactic acid feeding. Overall, the LAB-AAB co-culture model developed here offers a promising framework. It helps improve both the flavor and functional quality of citrus vinegar. It may also be extended to the fermentation of other fruit-based vinegars. This can promote the advancement of health-oriented fermented foods.

It is worth noting that although this study clarifies the regulatory role of inoculum size on acetoin accumulation and the core mechanism of over-oxidation, there are still certain limitations. For instance, the quantitative analysis of the dynamic distribution ratio of carbon sources between strains in the synergistic metabolism of the two strains is still lacking, and the molecular mechanism by which lactic acid produced by *Lactobacillus plantarum* NF2 precisely regulates the metabolic flux of *Acetobacter pasteurianus* NF171 toward acetoin synthesis remains to be further elucidated. Future studies could employ ^13^C isotope tracing technology to clarify the metabolic trajectory of carbon sources from glucose through lactic acid to acetoin. Meanwhile, combined metatranscriptomic and proteomic analyses could be used to identify key functional genes that regulate redox homeostasis (e.g., NADH/NAD^+^ balance), providing targets for constructing genetically engineered strains to enhance acetoin synthesis. In addition, regarding strategies to alleviate over-oxidation under high inoculum conditions, the feasibility of staged supplementation of antioxidants (such as glutathione) or dynamic pH adjustment to maintain redox balance during fermentation could be explored, offering more operable solutions for the stable accumulation of target products in industrial production.

## Data Availability

The datasets analyzed for this study can be found in KEGG online repositories (https://www.genome.jp/kegg/) and MagiGene Cloud Platform (http://cloud.magigene.com/).

## Footnotes

^1^ https://www.genome.jp/kegg/

## References

[ref1] Akasaka N. Sakoda H. Hidese R. Ishii Y. Fujiwara S. (2013). An efficient method using *Gluconacetobacter europaeus* to reduce an unfavorable flavor compound, acetoin, in rice vinegar production. Appl. Environ. Microbiol. 79, 7334–7342. doi: 10.1128/aem.02397-13, PMID: 24056455 PMC3837748

[ref2] Benziman M. Russo A. Hochman S. Weinhouse H. (1978). Purification and regulatory properties of the oxaloacetate decarboxylase of *Acetobacter xylinum*. J. Bacteriol. 134, 1–9. doi: 10.1128/jb.134.1.1-9.1978, PMID: 206534 PMC222210

[ref3] Chai L. Qiu T. Lu Z. Deng Y. Zhang X. Shi J.. (2020). Modulating microbiota metabolism via bioaugmentation with *Lactobacillus casei* and *Acetobacter pasteurianus* to enhance acetoin accumulation during cereal vinegar fermentation. Food Res. Int. 138:109737. doi: 10.1016/j.foodres.2020.109737, PMID: 33292931

[ref4] Chen L. Wang G. Teng M. Wang L. Yang F. Jin G.. (2023). Non-gene-editing microbiome engineering of spontaneous food fermentation microbiota—limitation control, design control, and integration. Compr. Rev. Food Sci. Food Saf. 22, 1902–1932. doi: 10.1111/1541-4337.13135, PMID: 36880579

[ref5] Cheng S. Li W. Yang H. Hou B. Hung W. He J.. (2025). Integrated transcriptomics and metabolomics reveal changes during *Streptococcus thermophilus* JM66 fermentation in milk: fermentation characteristics, flavor profile, and metabolic mechanism. Food Res. Int. 203:115770. doi: 10.1016/j.foodres.2025.115770, PMID: 40022315

[ref6] Chu C. Yu L. Li Y. Guo H. Zhai Q. Chen W.. (2023). *Lactobacillus plantarum* CCFM405 against rotenone-induced Parkinson’s disease mice via regulating gut microbiota and branched-chain amino acids biosynthesis. Nutrients 15:1737. doi: 10.3390/nu15071737, PMID: 37049578 PMC10096885

[ref7] Cui Z. Zheng M. Ding M. Dai W. Wang Z. Chen T. (2023). Efficient production of acetoin from lactate by engineered *Escherichia coli* whole-cell biocatalyst. Appl. Microbiol. Biotechnol. 107, 3911–3924. doi: 10.1007/s00253-023-12560-x, PMID: 37178309

[ref8] Deng M. Zhang S. Wu S. Jiang Q. Teng W. Luo T.. (2024). *Lactiplantibacillus plantarum* N4 ameliorates lipid metabolism and gut microbiota structure in high fat diet-fed rats. Front. Microbiol. 15:1390293. doi: 10.3389/fmicb.2024.1390293 38912346 PMC11190066

[ref9] Feng L. Xu J. Ye C. Gao J. Huang L. Xu Z.. (2023). Metabolic engineering of *Pichia pastoris* for the production of triacetic acid lactone. J. Fungi 9:494. doi: 10.3390/jof9040494, PMID: 37108948 PMC10145311

[ref10] Forde C. G. Cox A. Williams E. R. Boss P. K. (2011). Associations between the sensory attributes and volatile composition of cabernet sauvignon wines and the volatile composition of the grapes used for their production. J. Agric. Food Chem. 59, 2573–2583. doi: 10.1021/jf103584u, PMID: 21332199

[ref11] Gu P. Ma Q. Zhao S. Gao J. Li C. Zhou H.. (2022). Application of quorum sensing system in microbial synthesis of valuable chemicals: a mini-review. World J. Microbiol. Biotechnol. 38:192. doi: 10.1007/s11274-022-03382-6, PMID: 35978255

[ref12] Guo T. Kong J. Zhang L. Zhang C. Hu S. (2012). Fine tuning of the lactate and diacetyl production through promoter engineering in *Lactococcus lactis*. PLoS One 7:e36296. doi: 10.1371/journal.pone.0036296, PMID: 22558426 PMC3338672

[ref13] Han D. Yang Y. Guo Z. Dai S. Jiang M. Zhu Y.. (2024). A review on the interaction of acetic acid bacteria and microbes in food fermentation: a microbial ecology perspective. Foods 13:2534. doi: 10.3390/foods13162534, PMID: 39200461 PMC11353490

[ref14] Jiang B. Fang X. Fu D. Wu W. Han Y. Chen H.. (2022). Exogenous salicylic acid regulates organic acids metabolism in postharvest blueberry fruit. Front. Plant Sci. 13:1024909. doi: 10.3389/fpls.2022.1024909, PMID: 36388486 PMC9665327

[ref15] Jo Y. Han J. W. Min D.-L. Lee Y. E. Choi Y.-J. Lim S. (2015). Optimization of acetic acid fermentation for producing vinegar from extract of jujube (*Zizyphus jujuba* Mill.) fruits. Korean J. Food Sci. Technol. 47, 711–718. doi: 10.9721/kjfst.2015.47.6.711

[ref16] Kotik M. Javůrková H. Brodsky K. Pelantová H. (2021). Two fungal flavonoid-specific glucosidases/rutinosidases for rutin hydrolysis and rutinoside synthesis under homogeneous and heterogeneous reaction conditions. AMB Express 11:136. doi: 10.1186/s13568-021-01298-2, PMID: 34661772 PMC8523606

[ref17] Kuhn D. Schmitz C. Rama G. R. Costa M. A. De Gregorio P. R. Irazoqui J. M.. (2025). Exploring endogenous lactic acid bacteria potential: isolation to genetic insights on aromatic compounds. Food Microbiol. 131:104800. doi: 10.1016/j.fm.2025.104800, PMID: 40484521

[ref18] Li T. Wang X. Li C. Fu Q. Shi X. Wang B. (2023). Investigation of acid tolerance mechanism of *Acetobacter pasteurianus* under different concentrations of substrate acetic acid based on 4D label-free proteomic analysis. Foods 12:4471. doi: 10.3390/foods12244471, PMID: 38137274 PMC10742644

[ref19] Li L. Wei X. Yu W. Wen Z. Chen S. (2017). Enhancement of acetoin production from *Bacillus licheniformis* by 2,3-butanediol conversion strategy: metabolic engineering and fermentation control. Process Biochem. 57, 35–42. doi: 10.1016/j.procbio.2017.03.027

[ref20] Liu S. Laaksonen O. Kortesniemi M. Kalpio M. Yang B. (2018). Chemical composition of bilberry wine fermented with non-*Saccharomyces* yeasts (*Torulaspora delbrueckii* and *Schizosaccharomyces pombe*) and *Saccharomyces cerevisiae* in pure, sequential and mixed fermentations. Food Chem. 266, 262–274. doi: 10.1016/j.foodchem.2018.06.003, PMID: 30381185

[ref21] Nualkaekul S. Charalampopoulos D. (2011). Survival of *Lactobacillus plantarum* in model solutions and fruit juices. Int. J. Food Microbiol. 146, 111–117. doi: 10.1016/j.ijfoodmicro.2011.01.040, PMID: 21411170

[ref22] Özdemir G. B. Özdemir N. Ertekin-Filiz B. Gökırmaklı Ç. Kök-Taş T. Budak N. H. (2021). Volatile aroma compounds and bioactive compounds of hawthorn vinegar produced from hawthorn fruit (*Crataegus tanacetifolia* (lam.) pers.). J. Food Biochem. 46:e13676. doi: 10.1111/jfbc.13676 33650149

[ref23] Pang Z. Hao J. Li W. Du B. Guo C. Li X.. (2023). Investigation into spatial profile of microbial community dynamics and flavor metabolites during the bioaugmented solid-state fermentation of Baijiu. Food Biosci. 56:103292. doi: 10.1016/j.fbio.2023.103292

[ref24] Peterson B. W. Sharma P. K. van der Mei H. C. Busscher H. J. (2011). Bacterial cell surface damage due to centrifugal compaction. Appl. Environ. Microbiol. 78, 120–125. doi: 10.1128/aem.06780-11 22038609 PMC3255633

[ref25] Pudlik A. M. Lolkema J. S. (2010). Citrate uptake in exchange with intermediates in the citrate metabolic pathway in *Lactococcus lactis* IL1403. J. Bacteriol. 193, 706–714. doi: 10.1128/jb.01171-10 21115655 PMC3021216

[ref26] Qi Z. Yang H. Xia X. Wang W. Yu X. (2014). High strength vinegar fermentation by *Acetobacter pasteurianus* via enhancing alcohol respiratory chain. Biotechnol. Bioprocess Eng. 19, 289–297. doi: 10.1007/s12257-013-0727-0

[ref27] Qian J. Wang Y. Liu X. Hu Z. Xu N. Wang Y.. (2023). Improving acetoin production through construction of a genome-scale metabolic model. Comput. Biol. Med. 158:106833. doi: 10.1016/j.compbiomed.2023.106833, PMID: 37015178

[ref28] Qiu X. Yu L. Wang W. Yan R. Zhang Z. Yang H.. (2021). Comparative evaluation of microbiota dynamics and metabolite correlation between spontaneous and inoculated fermentations of Nanfeng tangerine wine. Front. Microbiol. 12:649978. doi: 10.3389/fmicb.2021.649978, PMID: 34046021 PMC8144288

[ref29] Rau M. H. Zeidan A. A. (2018). Constraint-based modeling in microbial food biotechnology. Biochem. Soc. Trans. 46, 249–260. doi: 10.1042/bst20170268, PMID: 29588387 PMC5906707

[ref30] Smid E. J. Kleerebezem M. (2014). Production of aroma compounds in lactic fermentations. Annu. Rev. Food Sci. Technol. 5, 313–326. doi: 10.1146/annurev-food-030713-092339, PMID: 24580073

[ref31] Smyth G. E. Orsi B. A. (1989). Nitroreductase activity of NADH dehydrogenase of the respiratory redox chain. Biochem. J. 257, 859–863. doi: 10.1042/bj2570859, PMID: 2494990 PMC1135667

[ref32] van Leeuwen P. T. Brul S. Zhang J. Wortel M. T. (2023). Synthetic microbial communities (SynComs) of the human gut: design, assembly, and applications. FEMS Microbiol. Rev. 47:fuad012. doi: 10.1093/femsre/fuad012, PMID: 36931888 PMC10062696

[ref33] Vicente J. Ruiz J. Tomasi S. de Celis M. Ruiz-de-Villa C. Gombau J.. (2022). Impact of rare yeasts in *Saccharomyces cerevisiae* wine fermentation performance: population prevalence and growth phenotype of *Cyberlindnera fabianii*, *Kazachstania unispora*, and *Naganishia globosa*. Food Microbiol. 110:104189. doi: 10.1016/j.fm.2022.104189 36462811

[ref34] Walsh A. M. Leech J. Huttenhower C. Delhomme-Nguyen H. Crispie F. Chervaux C.. (2023). Integrated molecular approaches for fermented food microbiome research. FEMS Microbiol. Rev. 47:fuad001. doi: 10.1093/femsre/fuad001, PMID: 36725208 PMC10002906

[ref35] Wang Q. Bao T. Hu M. Xu M. Rao Z. Zhang X. (2025). Efficient acetoin production in *Bacillus subtilis* by multivariate modular metabolic engineering with spatiotemporal modulation. ACS Sustain. Chem. Eng. 13, 1927–1936. doi: 10.1021/acssuschemeng.4c06511

[ref36] Wätjen A. P. Øzmerih S. Shetty R. Todorov S. K. Huang W. Turner M. S.. (2023). Utilization of plant derived lactic acid bacteria for efficient bioconversion of brewers' spent grain into acetoin. Int. J. Food Microbiol. 406:110400. doi: 10.1016/j.ijfoodmicro.2023.110400, PMID: 37742345

[ref37] Wei Y. Geng Q. Liu H.-P. Wang Y.-Q. Zhang G.-F. Qian X.-L.. (2024). Hierarchical engineering of meso-diaminopimelate dehydrogenase for efficient synthesis of bulky d-amino acids. ACS Catal. 14, 11447–11456. doi: 10.1021/acscatal.4c03164

[ref38] Xia M. Zhang X. Xiao Y. Sheng Q. Tu L. Chen F.. (2022). Interaction of acetic acid bacteria and lactic acid bacteria in multispecies solid-state fermentation of traditional Chinese cereal vinegar. Front. Microbiol. 13:964855. doi: 10.3389/fmicb.2022.964855, PMID: 36246224 PMC9557190

[ref39] Xu A. Xiao Y. He Z. Liu J. Wang Y. Gao B.. (2022). Use of non-*Saccharomyces* yeast co-fermentation with *Saccharomyces cerevisiae* to improve the polyphenol and volatile aroma compound contents in Nanfeng tangerine wines. J. Fungi 8:128. doi: 10.3390/jof8020128, PMID: 35205881 PMC8875693

[ref40] Yang T. Rao Z. Hu G. Zhang X. Liu M. Dai Y.. (2015). Metabolic engineering of *Bacillus subtilis* for redistributing the carbon flux to 2,3-butanediol by manipulating NADH levels. Biotechnol. Biofuels 8:129. doi: 10.1186/s13068-015-0320-1, PMID: 26312069 PMC4549875

[ref41] Yu Y. Ye H. Wu D. Shi H. Zhou X. (2019). Chemoenzymatic quantification for monitoring unpurified polysaccharide in rich medium. Appl. Microbiol. Biotechnol. 103, 7635–7645. doi: 10.1007/s00253-019-10042-7, PMID: 31372704

[ref42] Yuan Y. Hou X. Lei Y. Zhang Q. Wang D. Hu K.. (2025). Impact of compound fungal bran Qu fortification on Sichuan bran vinegar fermentation and product quality. Food Chem.:X. 28:102611. doi: 10.1016/j.fochx.2025.102611, PMID: 40520704 PMC12167117

[ref43] Zhang M. Li X. Mu D. Cai J. Zhang M. Liu Y.. (2022). Co-fermentation metabolism characteristics of apple vinegar with *Acetobacter pasteurianus* and *Lactobacillus plantarum*. J. Food Process. Preserv. 46:e16605. doi: 10.1111/jfpp.16605

[ref44] Zhao H. Wang Y. Wu Y. Kang X. Sam F. E. Hu K.. (2024). Impacts of non-*Saccharomyces* yeasts on nutrient composition and aroma profile of wines during co-fermentation with *Saccharomyces cerevisiae* and *Levilactobacillus brevis*. J. Food Compos. Anal. 136:106743. doi: 10.1016/j.jfca.2024.106743

[ref45] Zheng M. Cui Z. Zhang J. Fu J. Wang Z. Chen T. (2023). Efficient acetoin production from pyruvate by engineered *Halomonas bluephagenesis* whole-cell biocatalysis. Front. Chem. Sci. Eng. 17, 425–436. doi: 10.1007/s11705-022-2229-0

[ref46] Zuljan F. A. Repizo G. D. Alarcon S. H. Magni C. (2014). α-Acetolactate synthase of *Lactococcus lactis* contributes to pH homeostasis in acid stress conditions. Int. J. Food Microbiol. 188, 99–107. doi: 10.1016/j.ijfoodmicro.2014.07.017, PMID: 25100661

